# VEXAS Syndrome—Review

**DOI:** 10.1055/s-0043-1770958

**Published:** 2023-07-10

**Authors:** Yue Zhang, Xifeng Dong, Huaquan Wang

**Affiliations:** 1 Department of Hematology, General Hospital, Tianjin Medical University, Tianjin, China

**Keywords:** VEXAS syndrome, UBA1, vacuoles, autoinflammatory, hematological disorders

## Abstract

VEXAS (vacuoles, E1 enzyme, X-linked, autoinflammatory, somatic) syndrome is a newly defined refractory adult-onset autoinflammatory syndrome caused by somatic mutations in the ubiquitin-like modifier-activating enzyme 1 (UBA1) gene in hematopoietic stem and progenitor cells, resulting in a shift in UBA1 isoform expression. Thus, patients develop a spectrum of systemic inflammatory manifestations and hematologic symptoms. To date, patients respond poorly to immune suppressive drugs, except high-dose glucocorticoids, and no treatment guidelines have been established. Given the high mortality rate, VEXAS syndrome needs to be taken seriously by physicians in all specialties. This article aims to describe the key features, pathogenesis, and clinical manifestations of VEXAS syndrome to better understand the targeted treatment and improve the prognosis of VEXAS syndrome.

## Introduction

VEXAS (vacuoles, E1 enzyme, X-linked, autoinflammatory, somatic) syndrome was originally proposed in 2020 by Beck et al. They identified somatic mutations in myeloid-restricted ubiquitin-like modifier-activating enzyme 1 (UBA1) gene methionine-41 (p. Met41) in hematopoietic progenitor cells using Sanger sequencing in 25 male patients with systemic inflammatory manifestations and/or hematologic disorders. 1 The UBA1 gene encodes ubiquitin-activating enzyme 1, which is critical in initiating ubiquitylation, a process in which misfolded proteins in cells are labeled for degradation, and UBA1 mutations lead to reduced protein degradation and activation of autoimmune pathways, resulting in an uncontrollable inflammatory response. 1 2 3 The age range for the diagnosis of VEXAS syndrome is 43 to 87 years, indicating a late onset of the disease or a lack of early diagnosis. 4

## Key Features

VEXAS is an acronym for vacuoles, E1 enzyme, X-linked, autoinflammatory, and somatic: V acuoles are mainly found in myeloid and erythroid precursor cells; UBA1 is an X -linked gene encoding an ubiquitin-activating enzyme ( E1 enzyme) whose myeloid lineage-restricted s omatic mutations cause a utoinflammatory manifestations. 5

### Vacuoles

The characteristic vacuolation of myeloid and erythroid precursors on bone marrow specimens can be found in almost all patients with VEXAS syndrome. 1 6 Vacuoles are also occasionally found in eosinophils, monocytes, megakaryocytes, and plasma cells, but are usually absent in mature lymphocytes and fibroblasts, which may be related to the localization of the UBA1 somatic mutation. 1 6 7 However, the lack of vacuoles in the bone marrow smears of two patients with atypical UBA1 splice site mutations suggests that it is not a criterion for excluding the diagnosis of VEXAS syndrome. 8 9 Cytoplasmic vacuoles can also be found in other disorders such as acute alcoholism, copper deficiency, zinc toxicity, and myelodysplastic syndrome (MDS), suggesting that vacuoles are also not a specific criterion for the diagnosis of VEXAS syndrome. 10 11 12 13 Lacombe et al identified that the threshold of ≥ 10% of neutrophil precursors with > 1 vacuole was associated with VEXAS syndrome with excellent sensitivity and specificity. 3 Therefore, the UBA1 mutation should be further evaluated to assist in the diagnosis of VEXAS syndrome when vacuolation of hematopoietic precursor cells is found in patients, especially in older men with systemic inflammatory manifestations, at the same time, other possible causes of vacuolation have been excluded. Currently, vacuolar contents and the mechanism of cell vacuolation in patients with VEXAS syndrome are unclear.

### E1 Enzyme

The UBA1 gene encodes the ubiquitin-activating enzyme (E1 enzyme) which is the crucial enzyme of cellular ubiquitylation. 14 Ubiquitination is a protein posttranslational modification process for the degradation of misfolded proteins, in which ubiquitin-activating (E1), conjugating (E2), and ligating (E3) enzymes act sequentially. Ubiquitylation is essential for a variety of cellular processes, such as cell-cycle progression, deoxyribonucleic acid damage response, and immune signaling pathways. 10 Mutations in UBA1 lead to reduced ubiquitination and activation of autoimmune pathways, resulting in inflammatory manifestations. 1 2 13 15

### X-Linked

UBA1 is located on the X chromosome (Xp11.3) and can escape X chromosome inactivation. VEXAS syndrome is more common in older men, suggesting that women may be protected by the unmutated allele. 1 5 16 17 18 However, a few female patients with acquired X chromosome monosomy or structural deletion of the X chromosome have been reported. 19 20 21

### Autoinflammatory

The mechanism by which UBA1 defects lead to multiple autoinflammatory manifestations has not been clarified. Multiorgan autoinflammatory manifestations include fever, ear and nose chondritis, dermatological manifestations, pulmonary infiltrates, and vasculitis. Transcriptomic analysis of the patient's peripheral blood also confirmed the activation of multiple innate immune pathways. 1 22 It can be speculated that the reduction in cytoplasmic ubiquitylation caused by UBA1 mutation leads to the accumulation of unfolded proteins, which further activates the unfolded protein response and multiple inflammatory pathways, and an increased proportion of spliced XBP1 in monocytes was also reported, in addition, neutrophils may also be involved in exacerbating the inflammatory response by promoting the formation of neutrophil extracellular traps (NETs). 2 3 10 22 Beck et al found upregulated gene expression of proinflammatory cytokines in a zebrafish model with deletion of the UBA1 isoform homolog, suggesting that systemic inflammatory manifestations in patients with VEXAS syndrome may be associated with UBA1 mutations. 1

### Somatic

The UBA1 mutation in VEXAS syndrome is a somatic mutation, meaning that it is not present in all tissue cells. The minimum detection limit for somatic mutation by Sanger sequencing is 15 to 20%. 23 That is, variant allele frequencies (VAFs) below a threshold may be overlooked, so for a subset of patients, a more sensitive second-generation sequencing option is needed to improve detection rates. 24 However, it has been reported that high VAF of UBA1 detected by exon sequencing may be mistaken for germline mutations, which are associated with X-linked spinal muscular atrophy 2 (SMAX2), and this may cause bias in diagnosis or even affects subsequent treatment. 25 Sequencing of different tissues can be considered when the patient's clinical presentation is not consistent with SMAX2.

## Pathogenesis

Missense mutations in codon 41 of UBA1 were found in almost all patients with VEXAS syndrome, with the order of frequency being p.Met41Thr(c.122T > C), p.Met41Val(c.121A > G), and p.Met41Leu(c.121A > C). 1 26 27 28 These mutations result in reduced expression of the cytoplasmic UBA1b isoform (initiating translation at Met41) along with the formation of the catalytically impaired UBA1c isoform (initiating translation at the downstream start codon Met67), and this isoform switch correlates with loss of ubiquitination activity and activation of the innate immune pathway. 1 Several novel mutations have also been identified in a few patients with VEXAS syndrome: splice site variants at the junctions of intron 2 and exon 3 (c.118-2A > C, c.118-1G > C, c.119-1G > C), and c.167C > T, p. Ser56Phe which causes a temperature-dependent impairment of UBA1 catalytic activity rather than an alteration of the UBA1 isoform. 4 5 8 9 14 These mutations were lineage-restricted to myeloid cells in circulation. With the discovery of these atypical mutations, it is now hypothesized that the underlying mechanism of the disease may be contributed to rather a depletion of cytoplasmic UBA1b isoform than the production of UBA1c. 27 Different mutants have different effects on UBA1b expression levels, and a certain threshold of residual UBA1b expression is required for disease development, if too low, cannot cause clonal amplification. Some reporters concluded that the severity of VEXAS was negatively correlated with the translation of residual UBA1b. 27 29 Further research should aim to determine the minimum threshold of residual UBA1b expression as a causative factor, to elucidate the direct effect of reduced UBA1b on the functional state of cells, and even to consider restoring UBA1b expression as a therapeutic approach.

A growing number of studies have identified the cooccurrence of UBA1 mutations with other mutations in patients with VEXAS syndrome. Hage-Sleiman et al observed the clonal dynamics of the progressive replacement of the CALR (calreticulin) mutation associated with essential thrombocythemia by the UBA1 mutation in multiple bone marrow of patients, suggesting that the UBA1 mutation may compete with other mutations and progressively become dominant to show signs of VEXAS and that extensive screening for UBA1 is needed to confirm the diagnosis of VEXAS. 30 In a French cohort study, cooccurrence of mutant UBA1 with mutations in DNMT3A was found in 11/116 patients, both were high in VAFs. 26 At the same time, hematopoietic stem cells containing DNMT3A mutations were previously reported to be resistant to apoptosis and differentiation caused by inflammatory stress. 31 Thus, it is conceivable that acquired DNMT3A mutations may give UBA1 mutant cells a competitive survival advantage in the inflammatory environment of VEXAS syndrome. 32 33

## Clinical Description

VEXAS syndrome is a severe, progressive disease with multiorgan system involvement, manifested mainly by systemic inflammatory manifestations as well as hematological manifestations. Since it was first reported in 2020, the spectrum of clinical manifestations has gradually expanded with the rapidly increasing number of reported cases of VEXAS ( Table 1 ).

**Table 1:** Summary of characteristics of VEXAS patients' cohorts

	Beck et al, 2020 1	Bourbon et al, 2021 4	Obiorah et al, 2021 6	Tsuchida et al, 2021 23	van der Made et al, 2022 24	Georgin-Lavialle et al, 2022 26	Ferrada et al, 2022 27	Ferrada et al, 2021 39	Comont et al, 2022 54	Mekinian et al, 2022 55
Cases ( *n* )	25	11	16	14	12	116	83	13	11	12
Median age at onset (range)	64 (45–80)	66 (47–83)	57 (45–77)	72 (43.2–93.1)	69.5 (47–79)	67 (62.5–73)	66 (41–80)	56 (45–70)	61 (52–72)	75.5 (68–83)
Male sex, *n* (%)	25 (100)	11 (100)	16 (100)	12 (86)	12 (100)	111 (96)	83 (100)	13 (100)	11 (100)	12 (100)
Key features, *n* (%)										
Fever	23 (92)	10 (91)	14 (88)	8 (57)	10 (83)	75 (65)	69 (83)	13 (100)	10 (91)	1 (8)
Skin involvement	22 (88)	11 (100)	14 (88)	8 (57)	10 (83)	97 (84)	68 (82)	11 (85)	11 (100)	5 (42)
Rash			14 (88)							
Nodules	13 (52)	4 (36)	6 (40)			15 (13)				
Neutrophilic dermatosis	8 (32)		3 (20)			46 (40)				
Pulmonary infiltrate	18 (72)	5 (46)	13 (87)		8 (67)	47 (41)	47 (57)	10 (77)	6 (55)	2 (17)
Chondritis	16 (64)	5 (46)	11 (73)	14 (100)	5 (42)	42 (36)		13 (100)	5 (46)	4 (33)
Ear chondritis	16 (64)	5 (46)	11 (73)	12 (86)	5 (42)	37 (32)	45 (54)	13 (100)		
Nasal chondritis	12 (48)	1 (9)	7 (47)	5 (36)	2 (16)	18 (16)	30 (36)	12 (92)		
Thrombosis	11 (44)	4 (36)	10 (63)	2 (14)		41 (35)	34 (41)	8 (62)	7 (64)	1 (8)
Arthralgia or arthritis	17 (68)	11 (100)	7 (47)	4 (29)	4 (33)	33 (28)	48 (58)	6 (46)		5 (42)
Vasculitis		7 (64)			1 (8)	30 (26)				
Leukocytoclastic vasculitis	7 (28)		6 (40)		2 (16)					
Medium-vessel arteritis	3 (12)				1 (8)					
Gastrointestinal involvement	9 (36)				5 (42)	16 (14)			5 (46)	
Abdominal pain		1 (9)	4 (27)			10 (9)				
Diarrhea			2 (13)			8 (7)				
Neurological involvement	11 (44)				6 (50)	17 (15)			4 (36)	
Hearing loss	10/16 (63)	1 (9)	7 (47)				24 (29)	5 (50)		
Cochlea/vestibular dysfunction			1 (7)						1 (7)	
Ocular involvement	7 (28)	5 (46)		6 (43)	5 (42)	47 (40)	20 (24)	4 (32)	4 (36)	
Scleritis	1 (4)					10 (9)				
Episcleritis	3 (12)	1 (9)	2 (13)			14 (12)				
Iritis	1 (4)		1 (7)							
Macrocytic anemia	24 (96)	7 (64)	16 (100)	9 (64)	9 (75)		81 (97)			
Bone marrow vacuoles	18/18 (100)	11 (100)	16 (100)	7/8 (88)	6 (50)					
Classification criteria that were met										
Relapsing polychondritis	15 (60)	5 (46)		14 (100)	5 (42)		43 (52)	13 (100)		
Sweet's syndrome	8 (32)	5 (46)		1 (7)	2 (16)		18 (22)			
Polyarteritis nodosa	3 (12)	1 (9)								
Giant-cell arteritis	1 (4)		1 (7)							
MDS	6 (24)	6 (55)	6 (37.5)	6 (43)	4 (33)	58 (50)	26 (31)	3 (23)	11 (100)	12 (100)
Multiple myeloma or MGUS	5 (20)	1 (9)	4 (25)			12 (10)			2 (18)	
Laboratory finding										
CRP (mg/L)	73 (6.3–382)	114 (16.7–205)				61 (30–128)		17.7 (9.6–99.5)	30 (7.3–154)	
ESR (mm/h)	97 (22–> 140)					NR		66.5 (42–110)		
Genetic characteristics										
p. Met41Thr (c.122T→C)	15 (50)	5 (46)	8 (50)	3/8 (37.5)	7 (58)	52 (45)	50 (60)	8 (62)	3 (27)	
p. Met41Val (c.121A→G)	5 (20)	3 (27)	5 (31)	2/8 (25)	4 (33)	35 (30)	18 (22)	2 (15)	3 (27)	
p. Met41Leu (c.121A→C)	5 (20)	1 (9)	3 (19)	3/8 (37.5)	1 (8)	21 (18)	15 (18)	3 (23)	4 (36)	
Atypical splice-site mutations		2 (18)				8 (7)	0		1 (9)	
Cooccurring mutations										
DNMT3A	1 (4)		2 (13)		1 (8)	11 (9)			2 (18)	1 (8)
TET2						6 (5)			1 (9)	1 (8)
Current or past treatment										
Glucocorticoids	25 (100)	10 (90)		14 (100)	12 (100)	86 (74)			10 (90)	3 (25)
AZA		4 (36)			1 (8)	14 (12)			11 (100)	12 (100)
JAK inhibitor	5 (20)	3 (27)				15 (13)				
Anti-IL-1	13 (52)	1 (9)			7 (58)	19 (16)			3 (27)	6 (50)
TCZ	10 (40)	5 (46)		4 (29)	6 (50)				4 (36)	4 (33)
HSCT		1 (9)							3 (27)	
Deceased	10 (40)	3 (27)	8 (50)	5 (36)	6 (50)	18 (16)	20 (24)	3 (23)	1 (9)	1 (8)

Abbreviations: AZA, azacitidine; CRP, C-protein reactive; ESR, erythrocyte sedimentation rate; HSCT, hematopoietic stem cell transplantation; IL, interleukin; JAK, Janus kinase; MDS, myelodysplastic syndrome; MGUS, monoclonal gammopathy of undetermined significance; TCZ, tocilizumab; VEXAS, vacuoles, E1 enzyme, X-linked, autoinflammatory, somatic.

### Systemic Symptoms

#### Constitutional Symptoms

Recurrent fever is the most common presentation and was present in 74% of patients in some reported large cohorts. It may be accompanied by other discomforts such as weight loss, poor appetite and fatigue, and elevated acute-phase reactants could be found. 22 It may be a primary manifestation of VEXAS or a secondary manifestation of an acquired immunodeficiency following treatment with multiple immunosuppressive. 34

#### Dermatologic Manifestations

Skin manifestations were also one of the most common presentations, reported in up to 88% of the initial cohort. 1 The most common observed cutaneous presentations can be nonvasculitic (including neutrophilic dermatosis, erythema nodosum, Sweet's syndrome, pressure plaques, urticaria, injection site reactions, etc.) or vasculitic (such as leukocytoclastic vasculitis). Zakine et al identified the UBA1 mutation by sequencing and analyzing skin samples from VEXAS patients, the same as that found in myeloid cells. Therefore, the authors concluded that the dermal infiltration appears to be based on the clonal expansion of UBA1 mutant cells rather than on an inflammatory background. 35 36 Meanwhile, Lacombe et al found UBA1 mutations only in skin tissues from patients with neutrophilic dermatosis and not in other dermatoses (cutaneous leukocyte fragmentation vasculitis and spacer lipofuscinosis), possibly suggesting that UBA1 mutant clones are not present in nonneutrophilic dermatitis or that their clonal amount is too small to be detected. This leads to different therapeutic options: clearing the UBA1 mutation or blocking the inflammatory response. 37 Patients may first be seen in dermatology for skin lesions, so dermatologists must include VEXAS in the differential diagnosis when confirming skin disease and, if necessary, initiate genetic testing as early as possible to assist in the diagnosis of VEXAS.

#### Relapsing Polychondritis

More than 50% of patients already met the diagnostic criteria for relapsing polychondritis before the diagnosis of VEXAS was confirmed. Khitri et al found a higher incidence of fever (60%), skin lesions (82%), and pulmonary infiltrates (46%), a worse prognosis, a lower response rate to drugs, and a higher mortality rate in the VEXAS-RP group compared to the I-RP group. 38 A decision tree algorithm with near-perfect accuracy based on male sex, mean corpuscular volume > 100 fL, and platelet count < 200 k/μL may suggest that genetic testing for UBA1 mutation should be strongly considered to clarify VEXAS. 39

#### Systemic Vasculitis

VEXAS syndrome can cause inflammation of blood vessels of any size, including small, medium, and large vessel vasculitis. In the initial report, giant cell arteritis was confirmed in 1 of 25 patients (4%), which is a large vessel vasculitis. 1 Medium vessel vasculitis with polyarteritis nodosa and small vessel vasculitis with immunoglobulin A vasculitis and antineutrophil cytoplasmic antibody-associated vasculitis have been reported in rare cases. 10 As more and more cases are reported, it proves that we need to consider VEXAS as a differential diagnosis for refractory vasculitis with abnormal hematological manifestations. 40

#### Lung Involvement

Lung involvement, such as neutrophilic alveolitis, pleural inflammation, nonspecific interstitial pneumonia, cryptogenic organizing pneumonia, and bronchiolitis obliterans, is reported in up to 49% of patients with VEXAS syndrome. Chest computed tomography demonstrated diffuse bilateral ground-glass opacities and pleural effusions. 1 10 22

### Hematologic Aspects

#### Myelodysplastic Syndromes

Patients with VEXAS syndrome with concurrent MDS are described in different study cohorts. In a study cohort of 116 French VEXAS patients, MDS patients accounted for 50% (58/116). 26 One study reported that pancytopenia and myelodysplasia were observed in patients when severe systemic inflammation worsened but no longer after symptoms resolved, suggesting that bone marrow dysplasia and cytopenia by VEXAS syndrome are associated with severe systemic inflammation. 32 Mutations in the DNMT3A gene have been detected in some VEXAS patients and may be responsible for the coexistence of hematologic malignancies such as MDS in patients with VEXAS syndrome. 14 32 41 42 The current studies have hypothesized that the inflammatory environment caused by VEXAS favors the emergence of mutant clones and secondary MDS, or that VEXAS itself promotes MDS. 33 43 It has also been suggested that the DNMT3A mutation, which is closely related to MDS, may facilitate the amplification of VEXAS clones and, as mentioned above, even trigger more severe inflammatory manifestations. The causal relationship cannot be established yet. 5 44 The UBA1 mutation makes these patients more susceptible to MDS and other associated hematologic tumors than their counterparts, and therefore more likely to require close clinical follow-up. 13

#### Thrombotic Events

Thrombotic events may occur early in the course of VEXAS. The first description of VEXAS syndrome reported venous thromboembolism in 11 out of 25 patients (44%). 1 Obiorah et al reported a higher incidence of thrombotic complications in 10 out of 16 patients (63%) and even a small percentage of patients relapsed during anticoagulation therapy. 6 The incidence of venous thrombosis was much higher than that of arterial thrombosis, 34.4 and 1.6%, respectively. 45 Dysregulation of ubiquitination due to UBA1 somatic mutations has been proposed as a key driver of VEXAS thrombosis: chronic inflammation and abnormal release of multiple cytokines, enhanced spontaneous NET formation, and the presence of pathological antiphospholipid antibodies are among the factors that can cause platelet activation, abnormal hemostasis, and endothelial cell dysfunction, and even may promote the coagulation cascade. 6 45 46 This suggests that immunomodulation and immunosuppression may be considered as a strategy to prevent thrombotic events. However, a large study did not detect UBA1 mutations in 97 men over 50 years old with a first unprovoked thrombotic event, so the authors do not recommend systematic screening for UBA1 mutations in older adults with a first unprovoked thrombotic event, which may be overscreened. 46

Hematologic disorders in patients with VEXAS syndrome also include thrombocytopenia, macrophage activation syndrome, hemophagocytic lymphohistiocytosis, plasma cell disease, chronic lymphocytic leukemia, and progressive bone marrow failure. But no etiologic link between VEXAS and these disorders has been confirmed. 47

### Other Clinical Manifestations

Other rare clinical manifestations have been gradually reported, including ocular inflammation (periorbital edema, uveitis, scleritis), gastrointestinal infiltration (abdominal pain, chronic diarrhea, gastrointestinal bleeding), lymph node enlargement, arthritis (mostly affecting small joints of the ankle, knee, and hand), cardiac involvement (myocarditis, pericarditis), and neurological involvement (hearing loss, sensory neuropathy, acute attacks of chronic inflammatory demyelinating polyneuropathy, and even central nervous system involvement). 22 26 48

As a growing number of cases are reported, it has been suggested that the clinical manifestations of VEXAS syndrome are always associated with specific mutant genotypes: an unexplained inflammatory syndrome in P. Met41Val, inflammatory eye disease in P. Met41Thr, and Sweet syndrome in P. Met41Leu. A cohort observed a higher mortality rate and a significantly shorter median survival time for P. Met41Val than for other mutations. 26 27

Early recognition and diagnosis of the syndrome are challenging, and the diagnosis of VEXAS should be considered in all patients with refractory inflammatory disease with progressive hematologic abnormalities.

## Laboratory Parameters

Macrocytic anemia is found in almost all patients, along with normal folic acid and vitamin B12. 49 It is accompanied by thrombocytopenia, and reduced lymphocyte and monocyte counts. 13 43 50 Blood smears show increased erythrocyte volume, granulocytic anaplasmosis, and Pseudo-Pelger-Huët anomaly. Bone marrow smears show hypercellular marrow, a progressive increase in the myeloid: erythroid ratio (which may reflect disease progression), and characteristic cytoplasmic vacuolation of myeloid and erythroid precursor cells. 6 The anemia of inflammation usually presents as microcytic hypochromic anemia, so physicians should consider the possibility of VEXAS syndrome when macrocytic anemia is found in inflammatory diseases. 51

The analysis of the peripheral blood from patients with VEXAS syndrome showed highly activated inflammatory signatures in multiple pathways, including tumor necrosis factor (TNF), interleukin-6 (IL-6), and interferon-γ. Inflammatory markers, such as erythrocyte sedimentation rate and C-reactive protein, were significantly elevated in all patients. 1 13 An elevated antinuclear antigen, rheumatoid factor, and antiphospholipid antibodies were also observed in patients with VEXAS. 52

**Fig. 1:**
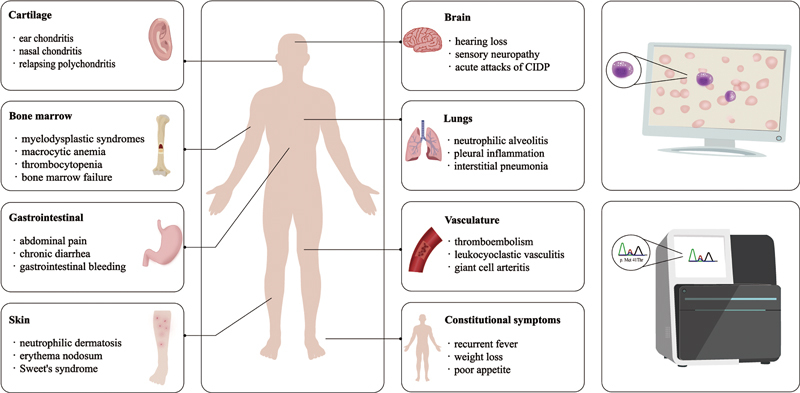
The clinical manifestations of VEXAS (vacuoles, E1 enzyme, X-linked, autoinflammatory, somatic) syndrome involve multiple organs and systems, such as systemic inflammatory manifestations and hematologic disorders, and characteristic cytoplasmic vacuoles are seen in almost all patients. Genetic sequencing is ultimately required to confirm the diagnosis of VEXAS syndrome.

Neither the complex, progressive clinical presentation nor the characteristic cytoplasmic vacuoles and abnormal laboratory tests can replace the detection of UBA1 mutations as criteria for confirming the diagnosis of VEXAS syndrome, and these nonspecific abnormalities can only be brought to the attention of physicians ( Fig. 1 ).

## Therapeutic Management

To date, there are no consistently effective therapies for VEXAS syndrome. Based on the current understanding of the pathophysiological mechanisms of VEXAS syndrome, two therapeutic strategies have been proposed: eradicating UBA1 mutations and blocking the inflammatory cascade response.

### Targeting and Eradicating UBA1 Mutated Cells

#### Hypomethylating Agents (Azacitidine)

Azacitidine (AZA) is the standard treatment for higher-risk MDS. Current studies have reported immunomodulatory and anti-inflammatory effects. 53 A cohort of VEXAS patients with concomitant MDS showed improvement in inflammatory symptoms after 4 cycles of AZA treatment in 5/11 (46%) patients. 54 A phase II prospective trial study found that 9/12 (75%) patients with UBA1 mutations achieved complete responses or partial responses after 6 cycles of AZA treatment, with improvement in inflammation-related manifestations, and sustainable steroid-sparing effects of AZA was observed, with the dosage below 10 mg/d. 55 In the small cohort ( *n* = 3) reported by Raaijmakers et al, three patients with VEXAS syndrome were treated with AZA and two of them were confirmed to have coexisting UBA1 and DNMT3A mutations; after receiving AZA, these two patients showed remission of inflammatory manifestations, a lower dosage of steroids, and an almost complete clearance of UBA1 and DNMT3A mutations in both bone marrow and peripheral blood was observed. The other patient who did not carry the DNMT3A mutation was unresponsive to AZA, which may indicate that VEXAS patients with DNMT3A mutation have increased sensitivity to AZA. 32 The poor efficacy of AZA found in the current study cohort may be due to the short duration of therapy, as hypomethylating agents usually works after 4 to 6 cycles in typical MDS. 56 The side effects are mild, and serious infections are common in patients with complex karyotypes and severely immunocompromised. 55 In the relatively limited sample size, the efficacy of AZA in VEXAS has not been proven and many questions remain: How does AZA improve inflammatory symptoms? Does AZA affect clonal amplification of UBA1 mutations, and is there a difference in sensitivity to AZA between VEXAS-MDS and VEXAS? 41

#### Hematopoietic Stem Cell Transplantation

Hematopoietic stem cell transplantation (HSCT) can remove abnormal bone marrow hematopoietic tissue through pretreatment and then implant healthy hematopoietic stem cells to rebuild the hematopoietic and immune systems. Six patients with VEXAS syndrome underwent allo-HSCT due to poor efficacy of glucocorticoids and multiple immunosuppressive drugs, severe inflammatory symptoms, or uncontrollable hematologic disease; three patients achieved complete remission, two patients survived during short-term follow-up, and one patient died of infectious complications following HSCT. 57 Another study reported a patient who was cured of MDS by autologous stem cell transplantation while clearing cells carrying the UBA1 mutation, suggesting that HSCT could be used as a curative option for VEXAS syndrome. 58 One study center reported that one patient received an early transplant after confirming VEXAS syndrome, which combined in vivo ATG-depleted T cells, in vitro depleted TCRαβ+ and CD19+ cells, and a myeloablative conditioning regimen (fludarabine, Marfarin, thiotepa), mescaline and prednisone to prevent graft-versus-host disease (GVHD). 15 This strategy in the absence of complications such as GVHD leads to successful transplantation. However, its indications and the prevention of complications still need attention. Specialists need to strike a balance between the potential for cure, the risk of complications, and recurrence. Several available assessment tools such as the Karnofsky Performance Scale score and Hematopoietic Cell Transplantation-Comorbidity Index can be used to screen suitable patients and assess their condition, then physicians need to determine the type of transplantation, the best preparative regimens, the specific timing of transplantation based on the actual condition, and protocols to deal with complications such as GVHD. If possible, long-term follow-up is recommended to evaluate the efficacy. 12 15 18 27 58 However, transplantation should be considered at an early stage, as VEXAS-related complications may limit the progression and efficacy of transplantation. A formal clinical trial to evaluate the efficacy of allogeneic stem cell transplantation in patients with VEXAS is underway. 59

### Targeting and Blocking the Inflammatory Cascade

#### Glucocorticoids

The available studies show that high doses of glucocorticoids are effective in ameliorating systemic inflammatory manifestations, while attempts to reduce the dose of glucocorticoids have resulted in poor symptom control and even recurrence. 1 47 52 60 61 Sustained application of glucocorticoids at high doses can lead to steroid dependence and serious adverse events (mainly infections and cardiovascular events). 12 Further research is needed on steroid-sparing drugs that work consistently.

#### Janus Kinase-Signal Transducer and Activator of Transcription Inhibitors (Ruxolitinib or Baricitinib)

Janus kinase (JAK) inhibitors prevent and treat a range of inflammatory and immune diseases by inhibiting JAK kinase and blocking the JAK-signal transducer and activator of transcription signaling pathway. The drug is effective against certain symptoms of systemic inflammatory diseases, especially skin involvement. 5 After applying ruxolitinib in place of AZA as monotherapy in one patient, significant regression of skin lesions was observed without an increasing dose of steroids, demonstrating the potential effectiveness of JAK inhibitors. 4 Heiblig et al found that complete clinical response and complete biological response were achieved in 7/11 (64%) and 6/11 (54%) patients treated with ruxolitinib after 1 month. 62 Subsequently, in their retrospective cases analysis, 87% of patients achieved complete clinical remission after 6 months of treatment with ruxolitinib, coinciding with significant increases in hemoglobin levels and platelet counts, reductions in steroids, and even discontinuation of steroids. 63 This multicenter study showed that the superiority of ruxolitinib over other JAK inhibitors may be attributed to its specific targeting of JAK1/JAK2, strong inhibitory activity against TYK2, and wide range of dose selection (with the possibility of gradually increasing dose). In addition, the clonal amplification of UBA1 was observed in patients, suggesting that the efficacy of ruxolitinib is related to its anti-inflammatory properties rather than to the eradication of UBA1 mutations. 30 63 However, side effects associated with JAK inhibitors are common, including severe opportunistic bacterial and viral infections including mycobacterium tuberculosis and herpes zoster virus, anemia, leukopenia, and thromboembolism. Infections and thromboembolic events can also occur in patients with VEXAS syndrome, making the safety of JAK inhibitors difficult to assess. 64 65 Given that the current treatment studies are retrospective analyses and lack control groups, there may be data selection bias, and prospective studies are still needed to further confirm their efficacy.

#### Tocilizumab

Tocilizumab (TCZ), a monoclonal antibody against the IL-6 receptor, may be effective in controlling systemic inflammation. Upregulation of expression of the gene encoding IL-6 in patients with VEXAS syndrome and its confirmation in a zebrafish model suggest that targeted therapy to block the IL-6 pathway may be an effective treatment. Goyal et al reported improvement in skin lesions after TCZ treatment in a patient with VEXAS syndrome, along with no need for blood transfusions and reduced steroid dose. 66 However, detailed reports following the TCZ administration are scarce to date. Within short-term follow-up, TCZ did partially alleviate inflammatory symptoms and reduce steroid dosage, but the long-term efficacy and side effects of TCZ are unclear. 4 47 67 68 69 70 Intestinal perforation is one of the severe comorbidities of TCZ; therefore, treatment with TCZ may increase the risk of intestinal perforation in patients with VEXAS who have underlying gastrointestinal involvement. 24 66

#### Anakinra and Canakinumab

Anakinra reduces the inflammatory response and relieves pain by blocking IL-1 signaling. Canakinumab selectively binds to IL-1β and blocks the interaction of IL-1β with its receptor. In one study, 1/6 (17%) patient was found to be responsive to anakinra, and 1/3 (33%) had severe local reactions at the subcutaneous injection site that prevented assessment of efficacy. 24 Campochiaro et al found that the combination of cyclosporine A and anakinra and canakinumab could be effective in improving inflammatory manifestations and also had steroid-sparing effects. However, neutropenia was found in 2/3 of patients. Perhaps more clinical trials and long-term follow-ups are needed to confirm its efficacy and safety. 71

Local injection site reactions to anakinra (usually mild to moderate, manifesting as redness, swelling, and pain) may be more severe in patients with VEXAS. Therefore, desensitization with intravenous anakinra may be considered in VEXAS patients to avoid side effects that limit the efficacy of anabolic agents. 72

### Others

Other immunosuppressive drugs used in VEXAS patients such as conventional synthetic disease-modifying antirheumatic drugs (methotrexate, mycophenolate mofetil, azathioprine), anti-TNF (adalimumab), calcineurin inhibitors (cyclosporine and tacrolimus), and abatacept were mostly effective temporarily during the limited follow-up period and may partially alleviate symptoms. 4 41 However, their efficacy needs to be confirmed in larger cohorts.

### Supportive Care

Long-term effective treatment options have not been established and treatment choices are still limited by retrospective study analysis. Therefore, patients may be given appropriate supportive therapy temporarily to improve their quality of life. For patients with anemia, erythropoiesis-stimulating agents are really important, and if necessary, individualized blood transfusion therapy can be considered, along with iron chelation therapy. 12 Patients with significantly reduced granulocytes are advised to prevent infection, and a granulocyte colony-stimulating factor may be administered. 12 It is currently recommended that patients with VEXAS should avoid coronavirus disease 2019 vaccination until its safety is assured. 73

Patients with thromboembolism may be treated with anticoagulation, and evaluation for the risk of bleeding and thrombosis is really necessary during treatment. There have been case reports of the successful use of apixaban in patients. Testing for antiphospholipid antibodies is also recommended to assist in the selection of vitamin K antagonists or oral anticoagulants. 18 45 Warfarin was previously reported to be more effective in patients with antiphospholipid syndrome. 74 In addition, multiple factors contribute to thrombosis, and it is conceivable that the use of immunosuppressive and immunomodulatory agents to modulate endothelial cell function and reduce the inflammatory milieu, combined with anticoagulation, could be a measure to avoid the recurrence of thrombotic events. Further studies are needed to clarify the mechanisms of thrombosis and to evaluate the efficacy of anticoagulation in patients with VEXAS. 45 Consideration of prophylactic anticoagulation in patients with VEXAS is still up in the air.

## Prognosis

From the available clinical data, patients with VEXAS have high clinical heterogeneity and mortality, with mortality rates of approximately 20 to 50% in different cohorts, and may have a poor outcome due to failure of early recognition and diagnosis, progression of hematologic disease, and lack of effective and efficient treatment modalities. Patients may die from disease or treatment-related complications.

Gastrointestinal involvement, pulmonary infiltration, mediastinal lymph node enlargement, and transfusion dependence are risk factors associated with high mortality. 26 The P. Met41Val mutant genotype was also a negative predictor of prognosis, and the inconsistency with the results of the French study cohort finding that patients with P. Met41Leu may have a better prognosis may be due to the shorter follow-up of the latter. The difference in survival between mutants may be due to their different levels of residual UBA1b translation. 27 No association between UBA1 VAF level and disease severity and mortality has been observed in the current study cohort. 26 27

## Conclusion

VEXAS syndrome is a recently identified refractory adult-onset inflammatory syndrome, mostly affecting elderly men. The main manifestations are systemic inflammation and hematologic manifestations, and no consistently effective treatment has been found except for high-dose glucocorticoids. Depending on the understanding of the molecular basis of the disease, it may be effective to target and eradicate UBA1 mutations (UBA1 gene editing to alter mutant clones, or HSCT to replace mutated progenitor cells), to block the inflammatory cascade response (targeting the ubiquitination pathway), or even to consider restoring the function of UBA1b. Bone marrow transplant and gene editing therapies may be a means of cure. Minimizing the time between the onset of symptoms of VEXAS syndrome and genetic diagnosis allows consideration of an allogeneic bone marrow transplant early in the disease. Therefore, testing for UBA1 mutations is recommended for adult patients with systemic inflammation, hematologic disorders, and vacuolation of myeloid and erythroid precursor cells to assist in the early diagnosis of the syndrome and to improve prognosis.
